# Assessment of White Matter Changes Using Quantitative T1ρ Mapping in an Open-Field Low-Intensity Blast Mouse Model of Mild Traumatic Brain Injury (mTBI)

**DOI:** 10.3390/ijms26125431

**Published:** 2025-06-06

**Authors:** Dina Moazamian, Shengwen Xie, Jiyo S. Athertya, Qingbo Tang, Roland R. Lee, Eric Y. Chang, Jeffrey M. Tomlin, Catherine E. Johnson, Jiang Du, Yajun Ma

**Affiliations:** 1 Department of Radiology, University of California, San Diego 9452 Medical Center, San Diego, CA 92037, USA; dmoazamian@health.ucsd.edu (D.M.); q1tang@health.ucsd.edu (Q.T.); rrlee@health.ucsd.edu (R.R.L.); e8chang@health.ucsd.edu (E.Y.C.); jiangdu@health.ucsd.edu (J.D.); 2 Radiology Service, VA San Diego Healthcare System, San Diego, CA 92161, USA; 3 Department of Neurological Surgery, University of California, San Diego, CA 92093, USA; jmtomlin@health.ucsd.edu; 4 Department of Explosive Engineering, Missouri University of Science and Technology, Rolla, MO 65409, USA; catherine.johnson@mst.edu; 5 Department of Bioengineering, University of California, San Diego, CA 92093, USA

**Keywords:** mTBI, open-field LIB, myelin loss, T1ρ, MRI

## Abstract

Blast-induced mild traumatic brain injury (mTBI) occurs when shock waves travel through blood vessels and cerebrospinal fluid, leading to cerebral demyelination, which results in cognitive impairments and neuropsychiatric issues that impact quality of life. This study aims to evaluate myelin changes in white matter in mice with mTBI induced by an open-field low-intensity blast (LIB) using a newly implemented 3D adiabatic T1ρ prepared fast spin echo (Adiab-T1ρ-FSE) sequence for quantitative T1ρ MRI mapping. Thirty male C57BL/6 mice, including 15 mTBI and 15 sham controls, were scanned on a 3T Bruker MRI scanner. Luxol fast blue (LFB) staining was performed to assess myelin content differences between the mTBI and sham control groups. A significantly higher T1ρ value in the medial corpus callosum (MCC) was found in mTBI mice compared to controls (126.8 ± 2.5 ms vs. 129.8 ± 2.5 ms; *p* < 0.001), consistent with the reduced myelin observed in LFB staining (0.80 ± 0.14 vs. 1.02 ± 0.06; *p* = 0.004). Moreover, a significant negative correlation between T1ρ and histological myelin content measurements was observed (r = −0.57, *p* = 0.02). Our findings demonstrate that T1ρ is a promising biomarker for detecting mTBI-associated demyelination in the brain.

## 1. Introduction

Mild traumatic brain injury (mTBI) caused by blast exposure is a significant concern, affecting both military personnel and civilians [1]. Blast waves travel through the cerebrospinal fluid and vasculature, leading to mechanical axonal damage and cerebral demyelination [2,3,4]. These pathological changes are associated with cognitive impairment, neuropsychiatric disorders, and behavioral deficits, which can significantly reduce quality of life [5,6,7,8,9]. Despite its clinical impact, diagnosing mTBI remains a major challenge due to the absence of immediate symptoms, lack of objective biomarkers, and the inability of conventional neuroimaging techniques—such as computed tomography (CT) and standard magnetic resonance imaging (MRI)—to detect subtle brain injuries [10,11]. Consequently, there is an urgent need for advanced imaging methods to improve diagnostic accuracy and track disease progression in mTBI patients [12,13].

MRI plays a pivotal role in ongoing research efforts to identify objective biomarkers of mTBI-related injury [14]. While conventional MRI techniques, such as T1- and T2-weighted fast spin echo (FSE) and T2 fluid-attenuated inversion recovery (FLAIR), are widely used in clinical settings, they lack the sensitivity to detect microstructural white matter (WM) changes following mTBI [15,16]. Various advanced MRI techniques—including diffusion tensor imaging (DTI), magnetization transfer ratio (MTR), susceptibility-weighted imaging (SWI), and proton magnetic resonance spectroscopy imaging (MRSI)—have demonstrated potential for detecting subtle mTBI-related abnormalities [17,18,19,20,21,22]. Recent studies have linked MRI-detected WM alterations to histopathological and proteomic markers of injury as well as to behavioral deficits [23,24,25,26]. Notably, changes in the corpus callosum (CC) have been consistently observed in blast-induced mTBI models, suggesting a potential imaging biomarker for injury assessment [14,27,28,29].

Despite these advancements, existing WM imaging techniques face critical limitations. Many require ultra-high field MRI (≥7 T), advanced hardware, and complex post-processing methods that are not widely available in clinical settings [30,31]. Furthermore, discrepancies in reported biomarker changes across studies—particularly in the same brain regions using identical MRI techniques—highlight the need for improved imaging approaches with greater reproducibility and sensitivity to microstructural damage [15,32,33,34,35].

One promising approach involves spin-lattice relaxation in the rotating frame, or T1ρ, which is sensitive to macromolecular changes in tissue [36,37,38,39]. This method has been successfully applied to assess pathological alterations in the cartilage [40], muscle [37], and liver [38], as well as those relating to autoimmune and neurodegenerative diseases [41,42,43]. For example, increased T1ρ relaxation times have been observed in the medial temporal lobe of Alzheimer’s disease patients compared to controls, suggesting sensitivity to early neurodegenerative processes [43]. The same group reported 10% higher T1ρ values in the hippocampus of patients with AD as compared to age-matched controls [42]. Given its ability to detect subtle macromolecular alterations, T1ρ imaging may provide valuable insights into WM pathology in mTBI.

However, traditional continuous wave-based T1ρ techniques are highly sensitive to B1 and B0 inhomogeneities, resulting in banding artifacts [44]. They are also sensitive to the magic angle effect, leading to significant variability in relaxation times depending on fiber orientation relative to the B0 field [45]. To overcome this limitation, adiabatic T1ρ (Adiab-T1ρ) techniques have been developed using adiabatic full passage (AFP) pulse trains to generate T1ρ contrast, reducing sensitivity to the magic angle and increasing robustness to B0 and B1 field inhomogeneities [46,47,48]. Adiab-T1ρ imaging has revealed abnormalities in neurodegenerative conditions such as Parkinson’s disease [49,50], central nervous system neoplasms [50], and stroke [51]. In animal studies, increased Adiab-T1ρ values have been linked to neuronal loss and WM degeneration, reinforcing its potential as a sensitive marker of microstructural damage [49,52].

This study aims to evaluate WM alterations in a murine model of mTBI induced by an open-field low-intensity blast (LIB) using a 3D Adiab-T1ρ-prepared FSE (Adiab-T1ρ-FSE) sequence [9,12,53,54]. T1ρ values in the medial corpus callosum (MCC) were measured and compared between mTBI and sham control groups. Histological staining was performed to validate MRI-detected WM alterations. By integrating advanced T1ρ imaging and histological analyses, this study aims to establish a more sensitive and clinically feasible approach for detecting microstructural WM changes in mTBI.

## 2. Results

**Figure 1:**
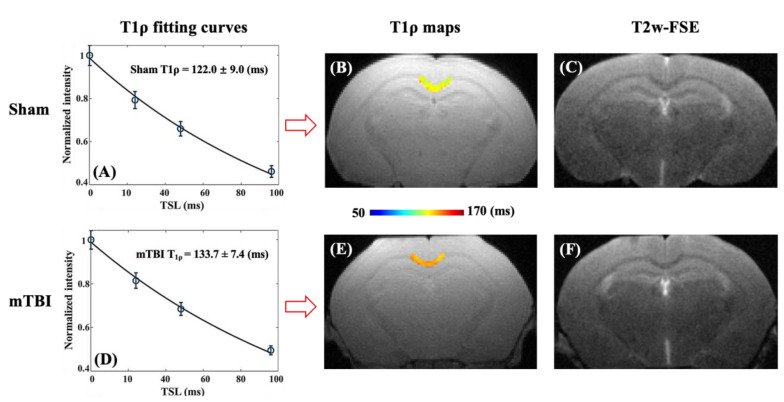
Representative T1ρ fitting curves (**A**,**D**) and pixel-wise T1ρ maps (**B**,**E**) as well as corresponding clinical T2-weighted fast spin echo (T2w-FSE) images (**C**,**F**) for an 8-week-old male sham control mouse (first row) and an mTBI mouse (second row). T2w-FSE images show no visible differences between sham control and mTBI mice. In contrast, the mTBI mouse exhibited a higher T1ρ value in the MCC compared to the sham control mouse (133.7 ± 7.4 ms vs. 122.0 ± 9.0 ms). MCC—medial corpus callosum; mTBI—mild traumatic brain injury; T2w-FSE—T2-weighted fast spin echo.

Figure 1 presents representative T1ρ fitting curves, pixel-wise T1ρ maps, and T2w-FSE images for a sham control and an mTBI mouse. No substantial differences were observed in clinical T2w-FSE images between the sham and mTBI mice. In contrast, the mTBI mice showed a higher mean T1ρ value in the MCC than the sham control mice (133.7 ± 7.4 ms vs. 122.0 ± 9.0 ms). This increase in T1ρ values suggests potential microstructural alterations in the WM following mTBI, which may not be detectable through conventional clinical MRI.

**Figure 2:**
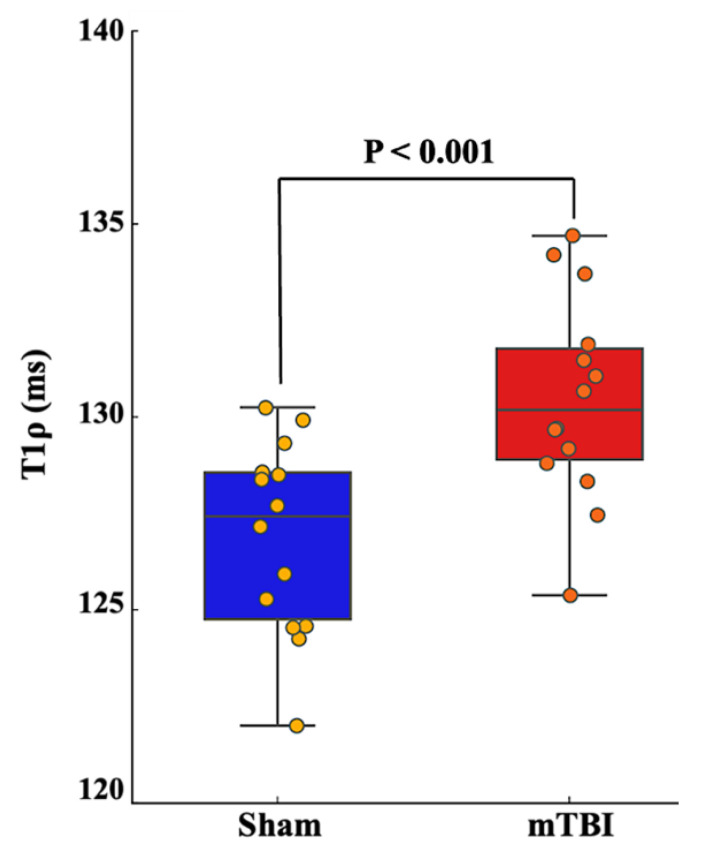
Bar-dot plot of T1ρ measurements comparing sham and mTBI groups. The mTBI group showed significantly higher T1ρ values than the sham group (129.8 ± 2.5 vs. 126.8 ± 2.5, *p* < 0.001, respectively). The edges of the box indicate the first and third IQR percentiles, respectively. mTBI—mild traumatic brain injury; IQR—interquartile range.

**Figure 3:**
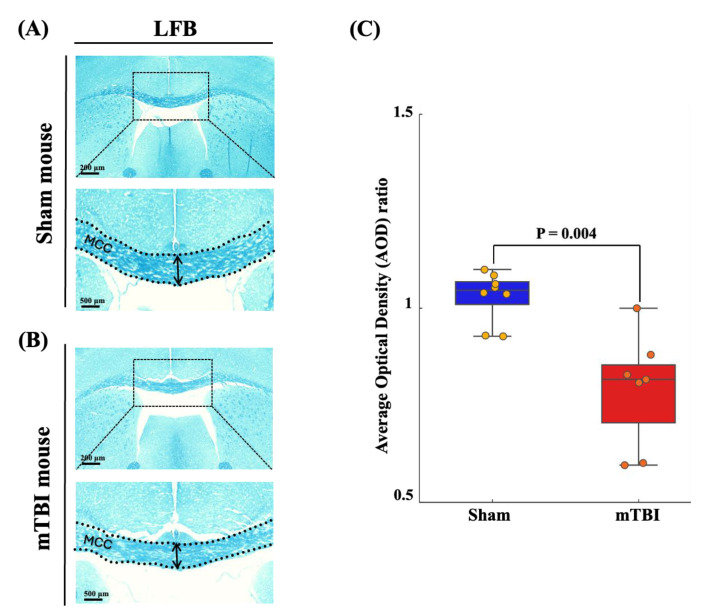
Representative brain LFB staining from a sham control mouse (**A**) and an mTBI mouse (**B**), along with a bar-dot plot of the AOD ratio measurements (**C**) in the MCC region for the sham and mTBI groups. The average AOD ratio in the MCC was significantly lower in mTBI mice compared to the sham group (0.80 ± 0.14 vs. 1.02 ± 0.06; *p* = 0.004) (**C**). The edges of the bar-dot plot box represent the first and third quartiles (IQR), respectively. LFB—Luxol Fast Blue; AOD—average optical density; MCC—middle corpus callosum, IQR—interquartile range.

Figure 2 presents bar-dot plots summarizing T1ρ values in the sham and mTBI groups in fourteen sham controls and fourteen mTBI. Two mice from each group were excluded from data analysis due to scanner malfunction. The mTBI group showed significantly higher mean T1ρ values compared to the sham controls (129.8 ± 2.5 ms vs. 126.8 ± 2.5 ms, *p* < 0.001), further supporting WM changes following mTBI. Figure 3 presents LFB staining images and quantification of myelin content in the MCC in eight sham controls and seven mTBI mice. The AOD ratio in the MCC is significantly lower in mTBI mice compared to sham controls (0.80 ± 0.14 vs. 1.02 ± 0.06, *p* = 0.004), indicating myelin loss associated with mTBI.

Supplemental Table S1 summarizes the average, standard deviation (SD), and statistical significance (independent *t*-test) of clinical T2w signal measures. The mTBI group shows no significant difference compared to the sham controls (2328 ± 305 vs. 2288 ± 353, *p* = 0.4).

Supplemental Figure S1A,B present bar-dot plots summarizing T1ρ values of the cortex and thalamus in the sham and mTBI groups. T1ρ values in the cortex were significantly elevated in the mTBI group compared to the sham controls (*p* = 0.02), indicating injury-related changes. In contrast, no significant differences were observed in the thalamus between groups (*p* = 0.98), suggesting regional specificity in the T1ρ alterations.

Supplemental Figure S1C,D present bar-dot plots summarizing AOD ratios of the cortex and thalamus in a random selection of four sham and four mTBI mice. The AOD revealed a significant reduction in myelin content in the cortex of mTBI mice (*p* = 0.007), consistent with T1ρ changes. In contrast, the thalamus showed no significant group difference in AOD values (*p* = 0.3), reinforcing the regional specificity of the observed alterations.

**Figure 4:**
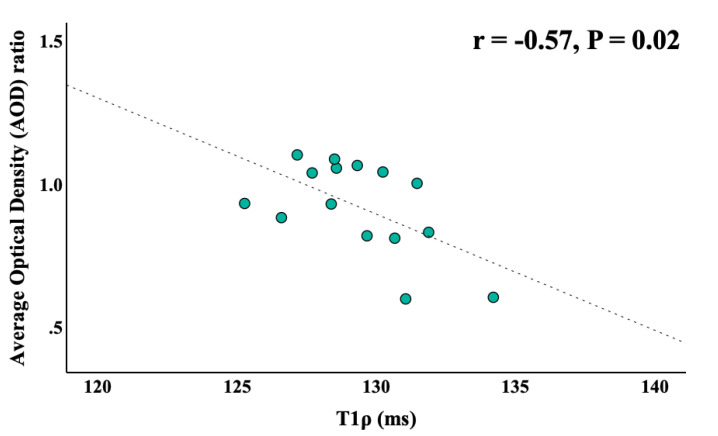
Correlation between T1ρ and AOD ratio measurements in eight sham controls and seven mTBI mice. A significant negative correlation was observed in the MCC region (*r* = −0.57, *p* = 0.02). LFB—Luxol Fast Blue; AOD—average optical density; MCC—middle corpus callosum.

The correlation between the MRI-measured T1ρ and the histology-measured AOD ratio is also investigated in this study. A significant negative correlation is found between T1ρ and AOD ratio values in the MCC (r = −0.57, *p* = 0.02) (Figure 4), indicating that higher T1ρ values correspond to a reduced myelin content. In addition, as shown in Supplemental Figure S2, a negative correlation was observed in the MCC between T1ρ and histological measures in the mTBI group alone (r = −0.43, *p* = 0.3). Although not statistically significant, this trend supports the hypothesis that increased T1ρ values reflect underlying myelin loss.

These results demonstrate that the 3D Adiab-T1ρ MRI technique is capable of detecting WM alterations in mTBI mice, as indicated by increased T1ρ relaxation times, histological evidence of myelin loss, and a significant correlation between MRI and histology findings.

## 3. Discussion

This study examined WM changes in mouse brains with mTBI induced by LIB using quantitative T1ρ imaging and histological staining. MRI analysis showed significantly higher T1ρ values in the MCC in mTBI mice compared to the sham group. The elevated T1ρ values suggest WM microstructural alterations, potentially linked to myelin integrity loss. Histological analysis supported this finding, showing significantly reduced myelin content in mTBI mice based on LFB staining. Further assessment of gray matter regions, including the cortex and thalamus, also showed a significant increase in the T1ρ values in the cortex, and there was no difference in the thalamus, which was consistent with the histology results that showed a significant reduction in the myelin content of the cortex and no significant difference in the thalamus between groups. These results highlight T1ρ imaging as a promising biomarker for assessing myelin loss in mTBI and monitoring disease progression over time.

Detecting the subtle structural changes associated with WM damage is challenging. Histology is considered the gold standard for evaluating mTBI pathology, but its use in human studies is ethically unfeasible. In clinical settings, conventional MRI and CT scans often fail to identify mild WM abnormalities, limiting their diagnostic utility [10,11]. Consequently, there is a growing need for more sensitive imaging biomarkers to detect acute mTBI and improve prognostic assessments.

The 3D Adiab-T1ρ-FSE sequence incorporates AFP pulses to enhance sensitivity to slow molecular motion in heterogeneous tissue environments. This unique feature allows for the in vivo investigation of molecular dynamics and the creation of distinct tissue contrasts in the MRI [36]. Additionally, by integrating the Adiab-T1ρ preparation with an FSE acquisition, this technique offers efficient data collection and is compatible with widely available MRI systems, making it a viable candidate for clinical translation. To the best of our knowledge, this is the very first study using Adiab-T1ρ to study mTBI.

The elevated T1ρ relaxation time observed in mTBI brains may reflect early neurodegenerative changes. This effect is likely driven by macromolecular breakdown in both intracellular and extracellular compartments, given that T1ρ relaxation is particularly sensitive to macromolecule–water interactions modulated by the applied radiofrequency field [21,39]. Similar adiabatic T1ρ or T2ρ MRI changes have been reported in neurodegenerative disorders, including Parkinson’s disease [32,33], CNS neoplasms [34], and stroke [35]. Notably, adiabatic T1ρ has been shown to detect neuronal loss in these conditions, further supporting its potential for identifying early pathological changes in mTBI [32,36].

The observed regional disparity in this study is likely due to the anatomical and biomechanical differences between cortical and subcortical structures. The cortex, being closer to the skull, is more directly exposed to blast wave forces, making it more susceptible to mechanical strain and microstructural disruption. In contrast, the thalamus is more centrally located and shielded by surrounding brain tissue, which may buffer it from the mechanical impact of mild blast waves [55,56].

In this study, ex vivo imaging was selected to avoid issues associated with in vivo scans, such as motion artifacts and physiological noise. The extracted brains were also positioned close to the surface coil to enhance signal-to-noise ratio (SNR) performance. This preliminary approach was designed to provide optimal image quality and accuracy for tracking myelin changes in the MCC. Although the technique is adaptable to in vivo applications, future work will focus on validating its use in vivo to assess its potential for clinical translation, especially for longitudinal studies.

This study is subject to a few limitations. First, a relatively small number of mice were used in this preliminary study. A higher number of mice should be examined in future pre-clinical studies. Second, potential factors that may impact T1ρ time constants could include inflammation, iron deposition, cell loss, microglial accumulation, and protein malformation. The sensitivity and specificity of T1ρ to myelin changes remain to be investigated in future studies. Third, this study has a variability in imaging timing (9–13 DPI), which may introduce subtle differences in injury progression; future studies should consider a narrower imaging window or longitudinal imaging for improved consistency.

Fourth, additional imaging modalities such as magnetization transfer ratio (MTR) or diffusion tensor imaging (DTI) were not included in this study, which could be explored and compared in future studies.

## 4. Methods and Materials

### 4.1. Animals

**Figure 5:**
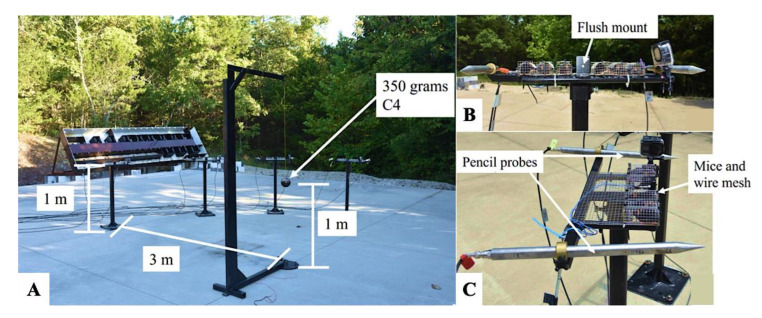
Experimental setup for the murine model of mTBI induced by open-field LIB exposure: (**A**) 350 g of C4 explosive placed 1 m above the ground and 3 m from the anesthetized mice, with the mice also positioned at 1 m in height. (**B**) Flush-mounted pressure sensor to measure head-on pressure. (**C**) Pencil probes to measure side-on pressure, with the mice housed in wire mesh to allow for unimpeded shock wave propagation. mTBI—mild traumatic brain injury; LIB—low-intensity blast.

A total of 30 male C57BL/6J mice, approximately eight weeks old, were obtained from Jackson Laboratories (Bar Harbor, ME, USA). The mice were housed in pairs in standard cages under a 12 h light/dark cycle with ad libitum access to food and water. They were randomly assigned to two groups: the mild traumatic brain injury (mTBI) group (*n* = 15) and the sham control group (*n* = 15). Repetitive open-field LIB exposure was performed at the open-air blast quarry of the Missouri University of Science and Technology using a well-established, highly reproducible murine LIB injury model [57] (Figure 5). Experimental outcome assessments were conducted in a double-blinded manner.

All experimental procedures adhered to the protocols approved by the Institutional Animal Care and Use Committees (IACUC) at the Harry S. Truman Memorial VA Hospital (protocol #1676549-5, approved on 28 July 2022) and the VA San Diego Healthcare System (protocol #1223488, approved on 7 July 2021). The study also followed the Animal Research: Reporting of In Vivo Experiments (ARRIVE) guidelines.

### 4.2. MRI Data Acquisition

Animals were transported to the VA San Diego Healthcare System for MRI scanning, conducted 10.8 ± 1.5 days post-injury (ranging from 8 to 13 days DPI). Before imaging, mice were anesthetized via an intraperitoneal injection of a ketamine/xylazine mixture (12.5 mg/mL ketamine, 0.625 mg/mL xylazine; 10 μL/g body weight). Once fully anesthetized, they were euthanized, and their brains were carefully extracted immediately before scanning.

MRI acquisition was performed using a 3T Bruker BioSpec system (Bruker, Billerica, MA, USA). A 10 mm receive-only surface coil was placed over the sample, while an 82 mm volume coil was used for signal transmission. Imaging was conducted utilizing the 3D Adiab-T1ρ-FSE sequence, optimized for high-resolution microstructural assessment, as well as with conventional 2D T2-weighted fast spin echo (T2w-FSE) for clinical evaluation.

**Figure 6:**
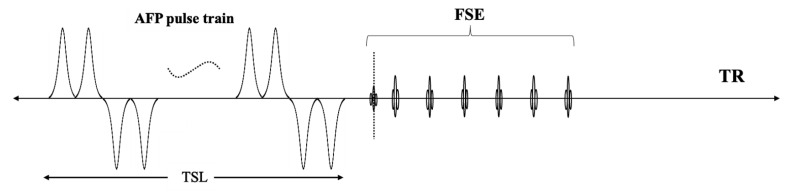
Diagram of the 3D Adiab-T1ρ-FSE sequence. Following a train of AFP pulses, a 3D FSE sequence is employed for rapid data acquisition. The AFP pulses lock the magnetization vector into a rotated frame, generating T1ρ contrast. 3D—three dimensional; FSE—fast spin echo; AFP—adiabatic full passage; TSL—time-of-spin-lock; TR—repetition time.

Figure 6 presents a schematic diagram of the 3D Adiab-T1ρ-FSE sequence, which integrates a train of AFP pulses followed by a 3D FSE readout for efficient data acquisition. The AFP pulses lock the magnetization vector into a rotated frame, generating T1ρ contrast and allowing for the characterization of the local macromolecule-water interaction system. The AFP pulse, shaped as a hyperbolic secant (HS1), has a duration of 6 ms and a peak amplitude of 17 µT [46,58].

The sequence parameters for mouse brain scans were as follows: (1) 3D Adiab-T1ρ-FSE: repetition time (TR) = 1500 ms, echo time (TE) = 5.5 ms, TSL = 0, 24, 48, and 96 ms (number of AFP pulses = 0, 4, 8, and 16), field of view (FOV) = 11 × 11 cm^2^, matrix size = 120 × 120, slice number = 40, slice thickness = 0.3 mm, and total scan time = 7 min; (2) 2D T2w-FSE: TR = 2699.6 ms, TE = 89.6 ms, FOV = 11 × 11 mm^3^, matrix size = 160 × 160, slice number = 16, slice thickness = 0.4 mm, and scan time = 8 min.

### 4.3. Histological Staining

Following MRI, brain samples were immediately fixed in zinc-formalin at 4 °C for 48 h, then paraffin-embedded and coronally sectioned at 5 μm thickness. Slides were collected from the Bregma −1.0 mm to −1.5 mm level at 50 μm intervals, corresponding to the MCC region. Three slides per sample were stained overnight in Luxol Fast Blue (LFB) at 60 °C, followed by brief differentiation with 0.05% lithium carbonate to enhance myelin contrast. Immunohistochemistry (IHC) using the proteolipid protein C1 (PLP-C1) antibody was conducted to assess myelin content in the cortex and thalamus. PLP-C1 was selected for gray matter staining because of its high sensitivity.

To ensure staining consistency across samples, consecutive sections from the same normal brain were processed as internal controls within each batch. The average optical density (AOD) ratio of the MCC, cortex, and thalamus was quantified using HALO AI software (v3.6.4134, Indica Labs, Albuquerque, NM, USA).

### 4.4. Data Analysis

For MRI data analysis, the MCC was selected as the region of interest (ROI) due to its high myelin density and susceptibility to traumatic forces, as previously reported by the field [58]. A single coronal slice per mouse was analyzed, with ROIs manually delineated based on anatomical landmarks. T1ρ measurements were obtained using a single-component exponential fitting model, and data were processed using MATLAB software, version R2023b (2023, The MathWorks Inc., Natick, MA, USA). A postdoctoral researcher, blinded to the information of the mice, performed ROI selection and image processing. The MCC was manually delineated using anatomical landmarks visible on clinical T2-weighted images, including the lateral ventricles, cingulate cortex, and caudate-putamen. In addition, to assess whether additional regions could provide further insights into the regional specificity of T1ρ changes, additional ROIs were manually defined in the cortex and thalamus. Mean T1ρ values were extracted from each ROI for statistical comparisons between groups.

Signal intensities from clinical T2w-FSE images were measured in the MCC to assess group differences. ROIs were manually delineated, and the average signal intensity values were calculated.

An independent *t*-test was used to compare T1ρ values and T2w-FSE signal intensities between the mTBI and sham groups.

For LFB-stained slides, the entire MCC region was included in the analysis. Since control slides within each batch originated from the same sample region, their AOD values were assumed to be consistent. To standardize staining intensity across batches, each control slide’s AOD was divided by the AOD of the first control slide in that batch, generating a normalization coefficient for inter-batch comparison. The PLP-C1 sections selected were imaged under identical conditions, and AOD values were quantified and compared for the cortex and thalamus of the four randomly selected sham control and four mTBI mice with an unpaired independent *t*-test.

The correlation between LFB staining and MRI measurements was assessed using Pearson’s test. SPSS 29.0 software (IBM, Armonk, NY, USA) was used for all statistical analyses. *p*-values less than 0.05 were considered statistically significant for all variables.

## 5. Conclusions

The T1ρ mapping technique enables the quantitative assessment of WM changes in the murine brain following open-field LIB-induced mTBI. Histological findings further validate the myelin loss in the MCC. The novel T1ρ imaging approach shows promise for evaluating mTBI-related WM alterations and holds potential for clinical translation.

## Data Availability

Data is contained within the article and Supplementary Materials.

## Supplementary Materials

The following supporting information can be downloaded at: https://www.mdpi.com/article/10.3390/ijms26125431/s1.

## Abbreviations

MRI, magnetic resonance imaging; mTBI, mild traumatic brain injury; LIB, low-intensity blast; CT, computed tomography; FSE, fast spin echo; FLAIR, fluid-attenuated inversion recovery; WM, white matter; DTI, diffusion tensor imaging; MTR, magnetization transfer ratio; SWI, susceptibility-weighted imaging; MRSI, proton magnetic resonance spectroscopy imaging; CC, corpus callosum; T1ρ, spin-lattice relaxation in the rotating frame; Adiab-T1ρ, adiabatic T1ρ; AFP, adiabatic full passage; CNS, central nervous system; MCC, medial corpus callosum; ROI, region of interest; TR, repetition time; TE, echo time; TSL, spin-lock duration time; FOV, field of view; AOD, average optical density; SPSS, Statistical Package for the Social Sciences; IACUC, Institutional Animal Care and Use Committees; ARRIVE, Animal Research: Reporting of In Vivo Experiments; DPI, days post-injury.
