# Supplementary material for: Assessment of White Matter Changes Using Quantitative T1ρ Mapping in an Open-Field Low-Intensity Blast Mouse Model of Mild Traumatic Brain Injury (mTBI)
Source: Int J Mol Sci. 2025 Jun 6;26(12):5431. doi: 10.3390/ijms26125431 (PMC12193592; doi:10.3390/ijms26125431)
Supplement: Supplementary file 1 [file ijms-26-05431-s001.zip › ijms-3567075-supplementary.pdf]

Supplemental Material

Supp.Table1. The average, standard deviation (SD), and their statistical significance (independent T-test) of T2w signal measures.

Groups	T2w Signal	T-test
**mTBI**	2328 ± 305	P = 0.4
**Sham**	2288 ± 353	

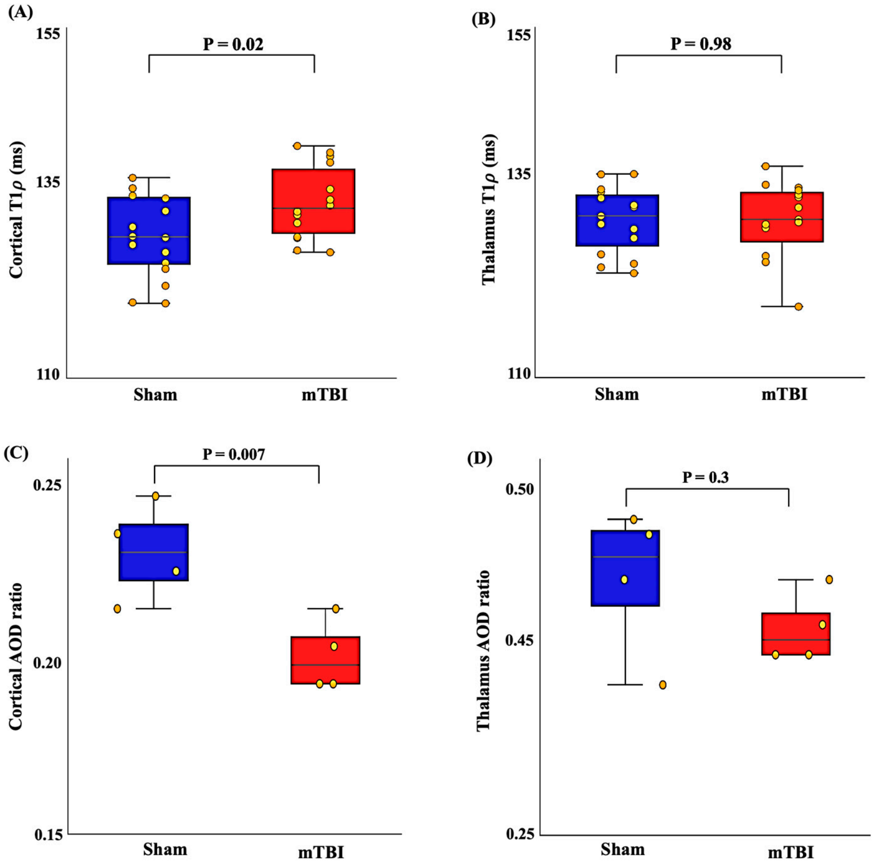

Supp. Figure 1. Box-dot plots of average optical density (AOD) ratios and T1ϱ measurements in the cortex and thalamus comparing sham and mTBI groups. The top plots show T1ϱ comparisons in the cortex (A, P = 0.02) and thalamus (B, P = 0.98) from. Bottom plots show AOD ratio comparisons for four randomly selected sham and four mTBI groups in the cortex (C, P = 0.007) and thalamus (D, P = 0.3). Significant differences were observed in both T1ϱ and AOD cortical measurements. No significant differences were found in the thalamus for either metric. The edges of the boxes represent the first and third interquartile range (IQR) percentiles.

mTBI, mild traumatic brain injury; AOD, average optical density; IQR, interquartile range.

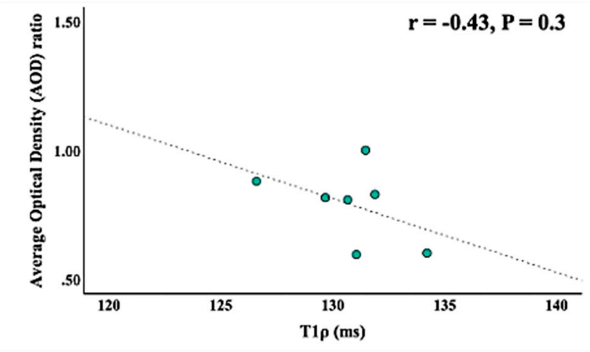

Supp. Figure 2. Correlation between T1ρ and AOD ratio measurements in seven mTBI mice. A negative correlation was observed in the MCC region, but it was not statistically significant (r = -0.43, P = 0.3).

mTBI, mild traumatic brain injury; AOD, average optical density; MCC, middle corpus callosum
